# Connective tissue disease-associated lung disease in children

**DOI:** 10.1007/s00247-024-05962-0

**Published:** 2024-06-08

**Authors:** Andrew H. Schapiro, Cara E. Morin, Kathryn A. Wikenheiser-Brokamp, Aki A. Tanimoto

**Affiliations:** 1 Department of Radiology, Cincinnati Children’s Hospital Medical Center, 3333 Burnet Avenue, Cincinnati, OH 45229 USA; 2 Department of Radiology, University of Cincinnati College of Medicine, Cincinnati, OH USA; 3 Department of Pathology and Laboratory Medicine, University of Cincinnati College of Medicine, Cincinnati, OH USA; 4 Division of Pathology & Laboratory Medicine, Cincinnati Children’s Hospital Medical Center, Cincinnati, OH USA; 5 Division of Pulmonary Medicine, Cincinnati Children’s Hospital Medical Center, Cincinnati, OH USA; 6 The Perinatal Institute Division of Pulmonary Biology, Cincinnati Children’s Hospital Medical Center, Cincinnati, OH USA

**Keywords:** Children, Computed tomography, Connective tissue disease, Lung, Pathology, Pediatric

## Abstract

Connective tissue diseases are a heterogeneous group of autoimmune diseases that can affect a variety of organ systems. Lung parenchymal involvement is an important contributor to morbidity and mortality in children with connective tissue disease. Connective tissue disease-associated lung disease in children often manifests as one of several radiologic-pathologic patterns of disease, with certain patterns having a propensity to occur in association with certain connective tissue diseases. In this article, key clinical, histopathologic, and computed tomography (CT) features of typical patterns of connective tissue disease-associated lung disease in children are reviewed, with an emphasis on radiologic-pathologic correlation, to improve recognition of these patterns of lung disease at CT and to empower the pediatric radiologist to more fully contribute to the care of pediatric patients with these conditions.

**Graphical abstract:**

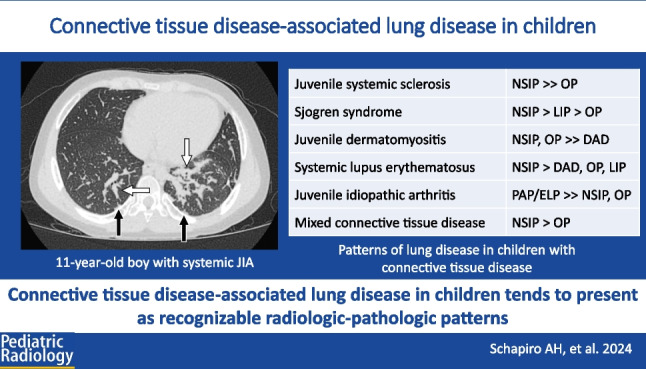

## Introduction

Connective tissue diseases represent a heterogeneous group of autoimmune diseases characterized by sustained inflammation, circulating autoantibodies, and organ damage [1, 2]. Lung parenchymal disease can occur in association with connective tissue disease in children, with certain lung disease patterns tending to occur in association with certain connective tissue diseases. The radiologist can play a valuable role in identifying and characterizing connective tissue disease-associated lung disease, tracking lung disease over time, suggesting the possibility of connective tissue disease in undiagnosed patients with appropriate imaging findings, and suggesting possible alternative or superimposed processes in patients with known connective tissue disease and abnormal lung findings on imaging.

We will first briefly discuss connective tissue disease-associated lung disease generally as well as the role of the radiologist in diagnosis and management of connective tissue disease-associated lung disease. We will then describe key clinical, histopathological, and computed tomography (CT) features of lung diseases seen in children with systemic sclerosis, Sjogren syndrome, juvenile dermatomyositis, systemic lupus erythematosus, juvenile idiopathic arthritis, and mixed connective tissue disease to familiarize the pediatric radiologist with this group of disorders.

## Connective tissue disease-associated lung disease

Several patterns of lung disease tend to be seen with connective tissue disease-associated lung disease including nonspecific interstitial pneumonia, organizing pneumonia, lymphoid interstitial pneumonia, diffuse alveolar damage, and a spectrum of pulmonary alveolar proteinosis and endogenous lipoid pneumonia. Because the interstitium is not the only portion of the lung involved in multiple of these pathologies, we refrain from using the often used terminology “interstitial lung disease” when describing these entities. While certain patterns of lung disease tend to occur in association with certain connective tissue diseases, more than one of these patterns can be associated with each connective tissue disease and many of the patterns can be seen with more than one connective tissue disease (Table 1). Although pediatric connective tissue disease-associated lung disease most often presents after a connective tissue disease diagnosis has already been made, it can occasionally be the presenting manifestation [3, 4].

**Table 1:** Relative frequency of lung disease patterns associated with connective tissue diseases

Juvenile systemic sclerosis	NSIP > > OP
Sjogren syndrome	NSIP > LIP > OP
Juvenile dermatomyositis	NSIP, OP > > DAD
Systemic lupus erythematosus	NSIP > DAD, OP, LIP
Juvenile idiopathic arthritis	PAP/ELP > > NSIP, OP
Mixed connective tissue disease	NSIP > OP

*NSIP* nonspecific interstitial pneumonia, *LIP* lymphoid interstitial pneumonia, *OP* organizing pneumonia, *DAD* diffuse alveolar damage, *PAP/ELP* pulmonary alveolar proteinosis/endogenous lipoid pneumonia spectrum

The specifics of these connective tissue disease-associated lung disease patterns will be described below, with each pattern discussed in more detail along with a connective tissue disease that it tends to be associated with so that each pattern is ultimately discussed in this article. In general, connective tissue disease-associated lung disease tends to be bilateral and relatively symmetric. In addition, preferential involvement of the anterior subpleural aspect of both upper lobes and involvement of the lower lobes is a disease distribution seen in some connective tissue disease-associated lung disease, particularly NSIP and pulmonary alveolar proteinosis/endogenous lipoid pneumonia spectrum, that is not typical of other lung diseases. This distribution has been noted in the adult connective tissue disease-associated lung disease literature as the “anterior upper lobe sign” and “four corners sign” and should prompt the radiologist to raise suspicion of connective tissue disease-associated lung disease, even in patients without known connective tissue disease [5, 6]. Additional thoracic findings associated with connective tissue disease including pleural and/or pericardial disease, airway disease, esophageal dilation, pulmonary artery enlargement, soft tissue calcifications, and joint abnormalities may also be present [7].

## Role of the radiologist

After selecting an appropriate CT protocol with which to image pediatric connective tissue disease-associated lung disease (Table 2), the radiologist can help determine prognosis and treatment at initial evaluation by determining whether connective tissue disease-associated lung disease is present in patients with known connective tissue disease, and if present, by determining the pattern and extent of disease [8–10]. After initial evaluation, the radiologist plays a vital role in evaluating for connective tissue disease-associated lung disease progression or response to treatment to direct therapy and further determine prognosis. In addition to identifying and characterizing connective tissue disease-associated lung disease in patients with known connective tissue disease, it is also important for the radiologist to recognize and suggest the possibility of connective tissue disease-associated lung disease in patients without known connective tissue disease, as lung disease can occasionally be the presenting manifestation [3, 4]. Finally, patients with connective tissue disease can be at increased risk for lung diseases other than connective tissue disease-associated lung disease, such as infection, edema, medication-induced pulmonary injury, aspiration, and lymphoproliferative disease. Thus, it is important for the radiologist to consider the possibility of alternative or superimposed processes in patients with connective tissue disease [1, 9, 11–13].

**Table 2:** Non-contrast chest CT protocol for evaluating connective tissue disease-associated lung disease

Scan	
Scan start/end location	Lung apices through lung bases
Breath hold	Inspiration
kVp	< 70 kg, 100
	> 70 kg, 120
mA	Dependent on patient size and automatic exposure control
	Minimum, 30 for smallest patients up to 80 for largest patients
	Maximum, 120 for smallest patients up to 260 for largest patients
Reconstructions	
Standard axial	< 15 kg, 3 mm
	> 15 kg, 5 mm
Lung axial	1 mm
Lung axial MIP	7 × 1 mm
Standard volume	0.5 × 0.3 mm
Coronal reformat	3 mm posterior to anterior
Sagittal reformat	3 mm left to right

*kVp* kilovoltage peak, *mA* milliamperes, *MIP* maximum intensity projection

## Juvenile systemic sclerosis

Juvenile systemic sclerosis is a rare heterogeneous connective tissue disorder characterized by vasculopathy, inflammation, and fibrosis of the skin and internal organs in patients younger than 16 years of age [14, 15]. The mean age of disease onset is 6–11 years old, with juvenile systemic sclerosis being very uncommon before the age of 5. Similar to adult systemic sclerosis, the pediatric disease is more common in females. Lung involvement is common, with a reported prevalence of 20–90%. Lung involvement, cardiac involvement, and pulmonary hypertension represent the leading causes of morbidity and mortality in patients with systemic sclerosis [15–19].

The most common histologic and CT pattern seen with systemic sclerosis-associated lung disease is nonspecific interstitial pneumonia (NSIP), which is highly associated with underlying connective tissue disease, most commonly systemic sclerosis [18, 20–22]. Additional thoracic imaging findings seen in the setting of juvenile systemic sclerosis include esophageal dilation with layering fluid, features of aspiration, pleural thickening or effusion, and features of pulmonary hypertension [18, 23].

### Nonspecific interstitial pneumonia

**Fig. 1:**
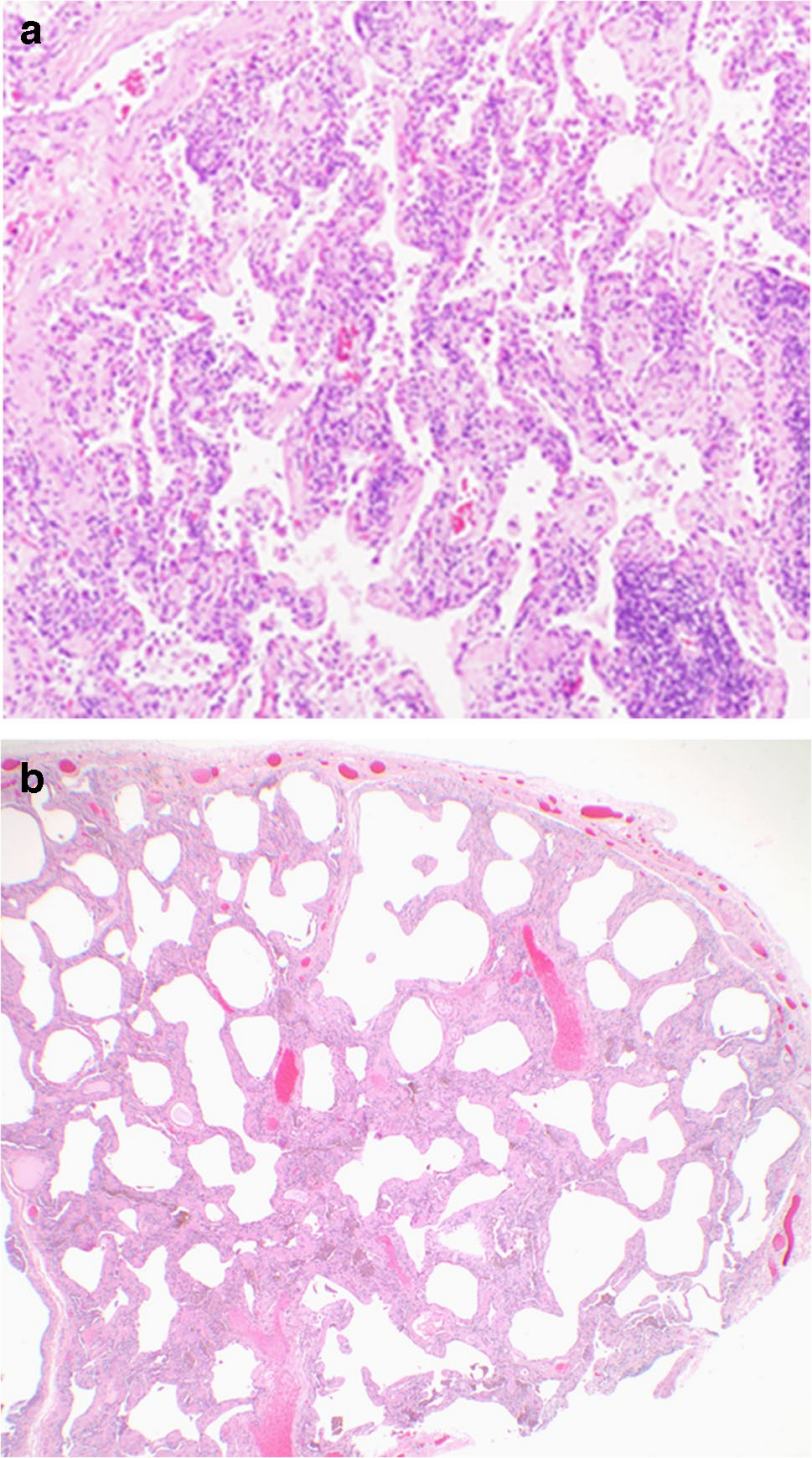
Nonspecific interstitial pneumonia (NSIP) histopathology. **a** Cellular NSIP with characteristic diffuse alveolar septal expansion by chronic inflammatory cells and minimal fibrosis (H&E, 100×). **b** Fibrotic NSIP with diffuse, temporally uniform alveolar septal expansion by fibrosis without significant architectural distortion, as well as mild chronic inflammation (H&E, 40×)

NSIP is histopathologically characterized by diffuse, varying amounts of interstitial inflammation and fibrosis with a temporally uniform appearance [24]. On one end of the spectrum, cellular NSIP is typified by diffuse chronic interstitial inflammation with absent or minimal fibrosis. While on the other end of the spectrum, fibrotic NSIP is characterized by diffuse, temporally uniform interstitial fibrosis with mild to moderate associated chronic inflammation. Both cellular and fibrotic NSIP lack substantial architectural distortion manifested as honeycomb change which, along with the temporal uniformity of lung injury and lack of conspicuous fibroblastic foci, differentiates NSIP from the temporally and spatially heterogeneous fibrosing entity, usual interstitial pneumonia [25–27] (Fig. 1).

**Fig. 2:**
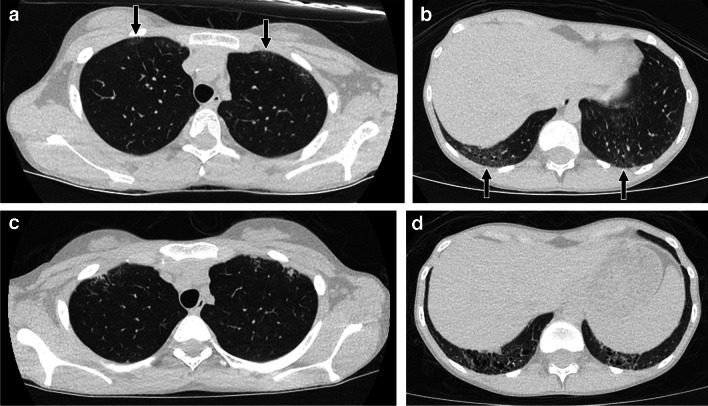
13-year-old female with systemic sclerosis. **a**, **b** Axial CT images without contrast showing subpleural ground-glass opacity, mild reticulation, and mild traction bronchiolectasis in the lower lobes and anterior upper lobes (black arrows) suggesting nonspecific interstitial pneumonia. **c**, **d** Follow-up axial CT images without contrast at 19 years of age showing increased reticulation and traction bronchiolectasis suggesting worsening fibrosis

The hallmark features of NSIP on CT include ground-glass opacity, accounted for by volume averaging of inflammation and/or fine fibrosis in the alveolar septa below the resolution of CT and air in the airspaces, as well as reticular opacities and traction bronchiectasis/bronchiolectasis that reflect areas of fibrosis [21]. The imaging findings tend to be lower lobe predominant, with a subpleural, peribronchovascular, or diffuse axial distribution. Subpleural anterior upper lobe involvement is also often seen in the setting of connective tissue disease-associated NSIP [6, 7] (Fig. 2). Subpleural sparing is not always present, but when seen is particularly suggestive of an NSIP pattern [10].

## Sjogren syndrome

Sjogren syndrome is a slowly progressive systemic autoimmune disease that primarily affects the exocrine glands, although other organs can also be affected [28, 29]. While Sjogren syndrome has been described as the second most common autoimmune connective tissue disease in adults, it is rarely diagnosed in the pediatric population, potentially due to under diagnosis [30–34]. In the pediatric population the mean age of onset is 10 years of age, and, similar to adults, there is a female preponderance [31, 32]. Unlike adults with Sjogren syndrome, children with Sjogren syndrome present more commonly with recurrent parotitis than sicca complex symptoms [31, 35]. Although extraglandular manifestations of Sjogren syndrome are common in pediatric patients with Sjogren syndrome, pulmonary involvement is extremely rare and may manifest as bronchial/bronchiolar and/or lung parenchymal abnormalities [33, 36–38].

NSIP is the most common pattern of lung disease seen in patients with Sjogren syndrome. However, lymphoid interstitial pneumonia (LIP) is also strongly associated with Sjogren syndrome and is most commonly seen in association with Sjogren syndrome as compared to other connective tissue diseases [28]. Although lung disease associated with Sjogren syndrome is extremely rare in children, both SS-associated NSIP and LIP have been reported in children [34, 37].

### Lymphoid interstitial pneumonia

**Fig. 3:**
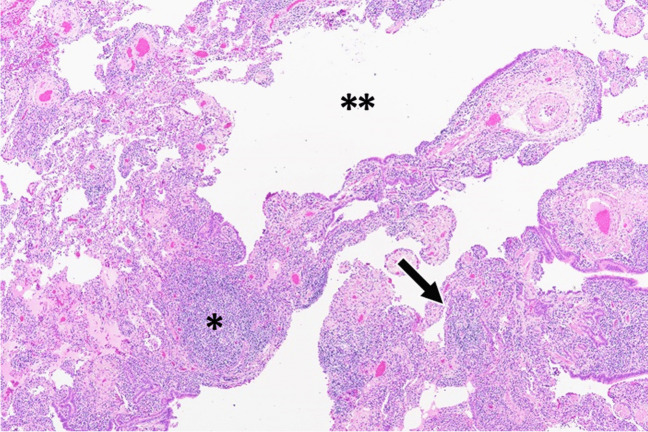
Lymphoid interstitial pneumonia histopathology. Characteristic dense cellular infiltrate expanding the alveolar septa (arrow) with a peribronchiolar lymphoid follicle (*) and an adjacent cystically dilated space (**) (H&E, 40×)

Histologically, LIP is characterized by a diffuse densely cellular interstitial infiltrate consisting mainly of polyclonal lymphocytes and plasma cells that widen the alveolar septa resulting in distortion of alveolar spaces [39–42] (Fig. 3). Lymphoid interstitial pneumonia is considered to be an entity in a spectrum of pulmonary lymphoproliferative disorders that includes follicular bronchiolitis. Accordingly, peribronchial and peribronchiolar lymphoid follicles with reactive germinal centers are often present along with the diffuse dense lymphocytic interstitial infiltrate and lymphoid follicles expanding alveolar septa that typify LIP [40, 43–45].

**Fig. 4:**
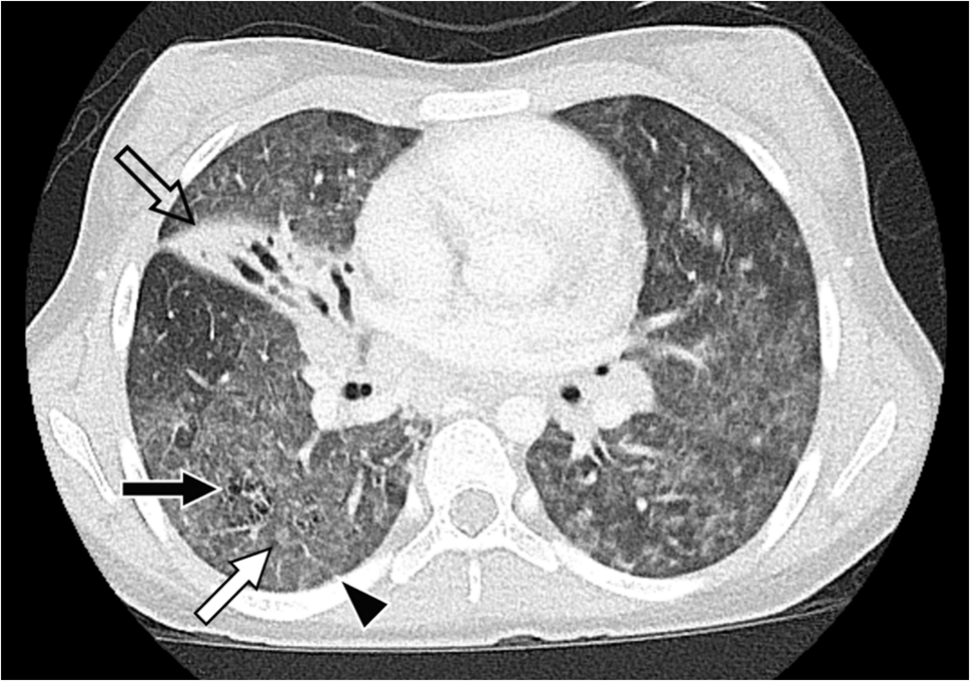
14-year-old female with Sjogren syndrome. Axial CT image with contrast showing extensive ground-glass opacity, peribronchovascular cysts (black arrow), ill-defined centrilobular nodules (white arrow), and interlobular septal thickening (arrowhead) suggesting lymphoid interstitial pneumonia. Right middle lobe collapse and bronchial dilation are also present (open arrow)

Similar to NSIP, the combination of inflammation expanding the alveolar septa and air within the alveolar spaces in LIP is below the resolution of CT, thus resulting in ground-glass opacity, the major finding of LIP on CT. Additional CT findings of LIP include lower lung predominant peribronchovascular and subpleural cysts that may relate to partial bronchiolar obstruction by peribronchiolar lymphocytic aggregates resulting in post-obstructive bronchiolar ectasia or overinflation. Interlobular septal and peribronchovascular thickening may also be present reflecting the propensity of the lymphocytic infiltrate to be most pronounced in the perilymphatic interstitium. Ill-defined centrilobular nodules are also described, corresponding to sites of more pronounced peribronchiolar lymphocytic infiltration [39, 41, 46–48] (Fig. 4).

## Juvenile dermatomyositis

Juvenile dermatomyositis is a systemic autoimmune vasculitis characterized by inflammatory myopathy and skin rashes. Although rare, juvenile dermatomyositis is the most common idiopathic inflammatory myopathy of childhood and presents as multiple subtypes and clinical courses [49–52]. There is a female preponderance of disease, and the average age of onset is 7 years [12, 53–55]. While cutaneous and muscular involvement is required for diagnosis, circulating autoantibodies may also result in lung disease. Indeed, restrictive lung disease is a rare but well-known complication of juvenile dermatomyositis, and assessment for lung disease is of particular importance given the significant associated morbidity and mortality [12, 52, 53, 56–58].

NSIP, organizing pneumonia (OP), and combined NSIP/OP features are the most common lung disease pathologic patterns seen in patients with dermatomyositis [12, 39, 56, 59]. Similar to NSIP, OP can be seen in the setting of numerous connective tissue disorders, but among the connective tissue diseases, OP is most commonly seen in association with dermatomyositis [28]. Diffuse alveolar damage (DAD) uncommonly occurs in association with juvenile dermatomyositis but is notable for its high mortality rate [60]. Aspiration resulting from pharyngeal-esophageal dysmotility and hypoventilation due to respiratory muscle dysfunction may also account for respiratory manifestations in patients with juvenile dermatomyositis [12].

### Organizing pneumonia

**Fig. 5:**
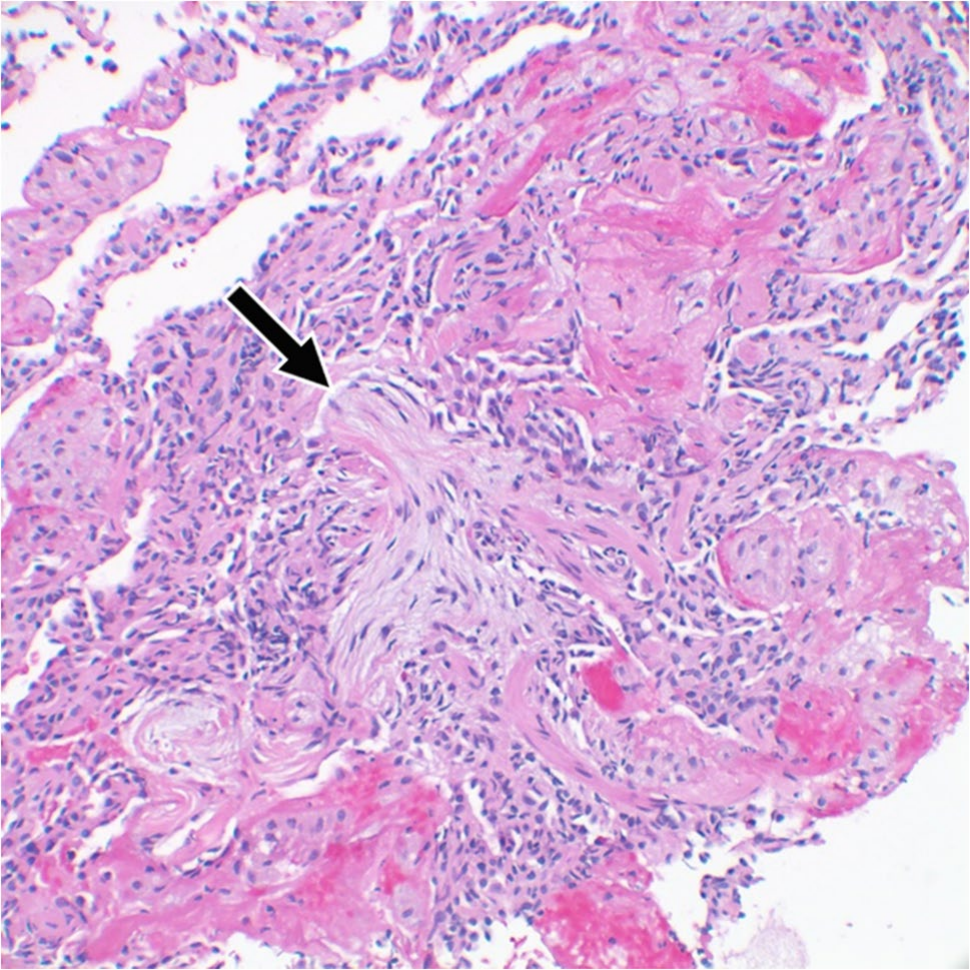
Organizing pneumonia histopathology. Branching fibroblastic tissue with a myxoid stroma filling distal airspaces (arrows) associated with a mild chronic inflammatory infiltrate characteristic of organizing pneumonia (H&E, 200×)

The hallmark histopathologic finding of OP is polypoid fibroinflammatory tissue filling distal bronchioles, alveolar ducts, and peribronchiolar alveoli with intact alveolar septa involved by mild interstitial inflammation [61–63] (Fig. 5).

**Fig. 6:**
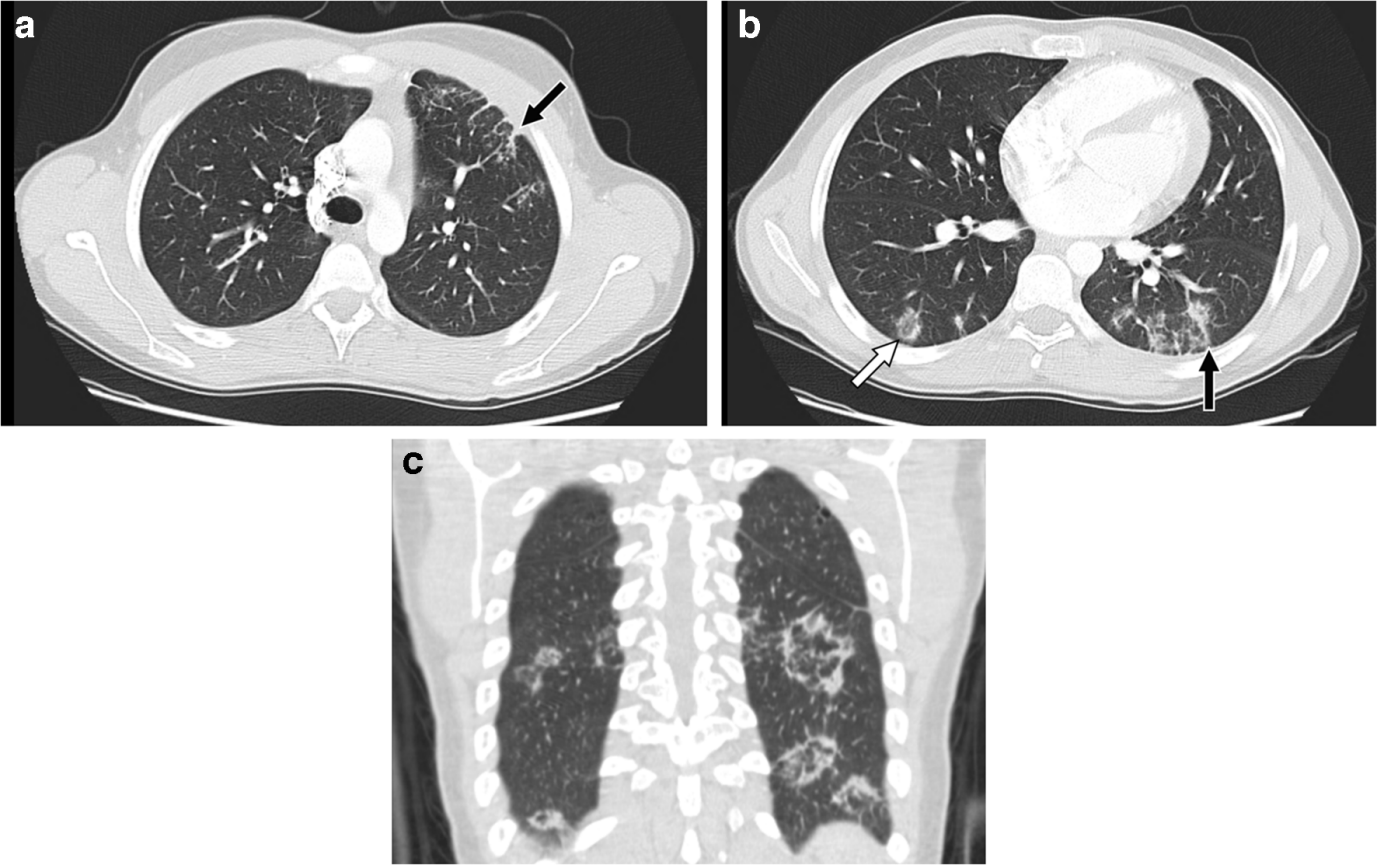
20-year-old male diagnosed with dermatomyositis when he was 17 years old. **a**, **b** Axial and (**c**) coronal CT images with intravenous contrast showing areas of peripheral perilobular opacity (black arrows) and reversed halo sign (white arrow) in the lower lobes and anterior left upper lobe suggesting organizing pneumonia

OP usually appears on CT as peribronchovascular and/or peripheral areas of consolidation with or without ground-glass opacity. The consolidation seen at imaging corresponds to dense plugging of small airways and alveolar spaces with fibroinflammatory tissue combined with alveolar septal thickening. The ground-glass opacity corresponds to alveolar septal thickening, intraluminal cellular debris, and lesser amounts of intraluminal fibroinflammatory tissue [61, 64–71]. Additional CT findings include perilobular opacities seen as subpleural predominant ill-defined curvilinear or polygonal opacities surrounded by aerated lung, and the “reversed halo”/“atoll” sign in which crescentic or ring-shaped consolidation surrounds areas of ground-glass opacity [65, 70–72] (Fig. 6). Single or multiple ill-defined nodular opacities can also be seen in the setting of OP, often in a peribronchial/peribronchiolar distribution with internal air bronchograms, but other findings of OP are usually also present [61, 73, 74]. Over time, CT findings of OP can completely resolve, although findings of fibrosis including reticulation, ground-glass opacity (reflecting fine fibrosis), parenchymal bands, architectural distortion, and/or traction bronchiectasis/bronchiolectasis, sometimes in an NSIP pattern, may remain at follow-up [68, 69, 73].

## Systemic lupus erythematosus

Systemic lupus erythematosus (SLE) is a severe, chronic, systemic autoimmune disease that impacts multiple organ systems [75]. Approximately 20% of cases are diagnosed during childhood, although very rarely before 5 years of age [76]. While adult-onset SLE is seen predominantly in females at a 13:1 ratio, significantly more males are diagnosed in the pediatric age group with an overall female-to-male ratio of 6:1 [76]. Typically, childhood-onset SLE has a more severe clinical phenotype than adult-onset SLE [75, 76]. Thoracic findings of SLE are common and include pleuritis and/or pericarditis with or without effusion, acute and chronic lung disease, shrinking lung syndrome, pulmonary arterial hypertension (PAH), and pulmonary embolism [77–79].

Among the acute lung diseases seen in the setting of SLE are “acute lupus pneumonitis,” which manifests histopathologically as DAD, and diffuse alveolar hemorrhage (DAH). Both of these severe pulmonary manifestations of SLE are rare but, importantly, are associated with a high mortality rate [77, 80, 81]. Chronic lung disease is uncommon in the setting of SLE, with NSIP being the most common pattern observed [77].

### Diffuse alveolar damage

**Fig. 7:**
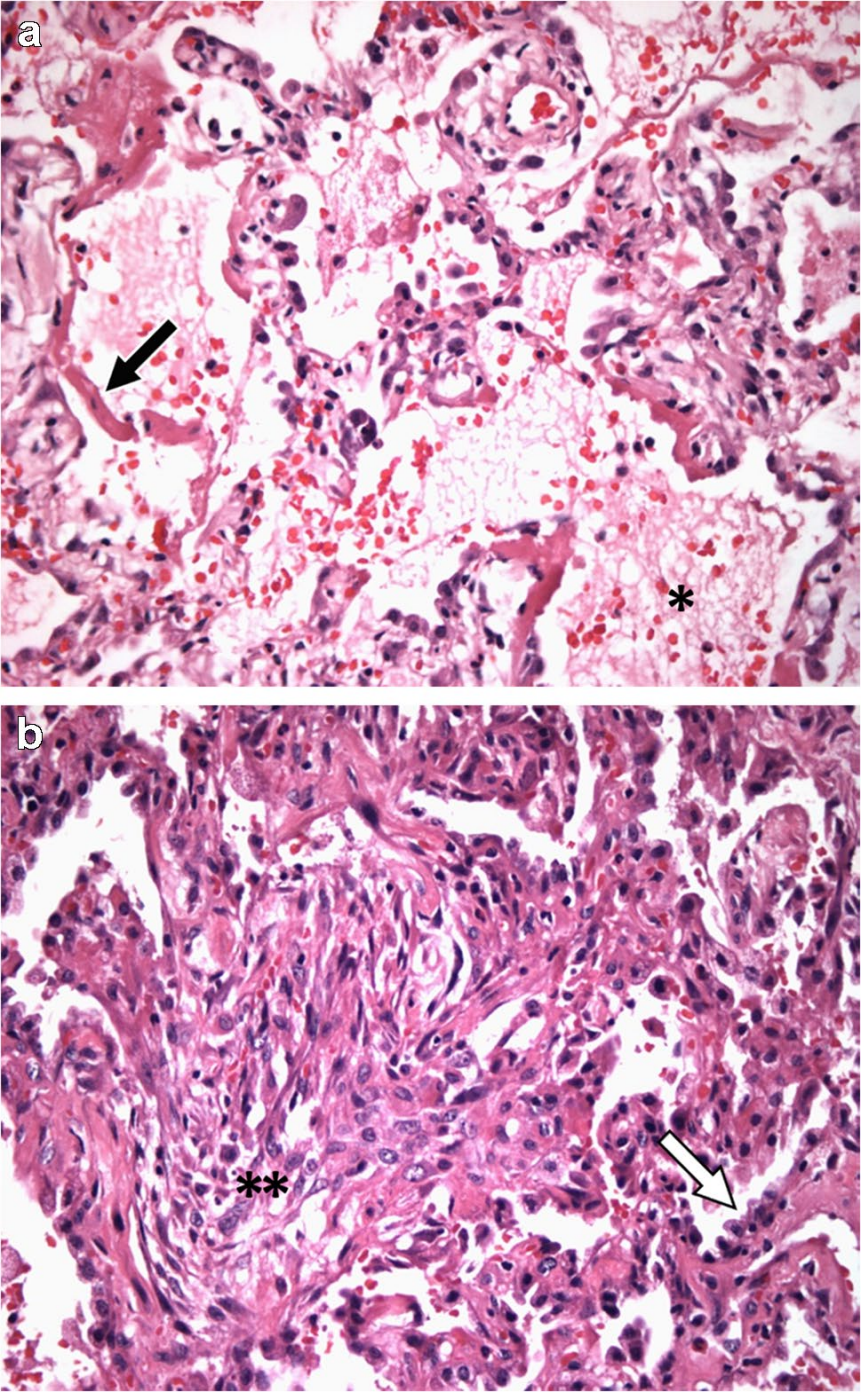
Diffuse alveolar damage (DAD) histopathology. **a** Acute DAD with hyaline membranes (black arrow) and intra-alveolar edema admixed with red blood cells (*) resulting from capillary injury (H&E, 400×). **b** Organizing DAD with interstitial fibrosis (**) and alveolar epithelial cell hyperplasia (white arrow) (H&E, 400×)

The histopathologic features of DAD are dependent upon the timing of tissue sampling after lung injury. In the first week or acute phase of lung injury, intra-alveolar and alveolar wall edema as well as hyaline membranes composed of cellular debris, plasma proteins, and surfactant predominate. Thrombi may also be present in small pulmonary arteries and capillaries. Starting in the second week or organizing phase of lung injury, fibroblast and alveolar epithelial type II pneumocyte hyperplasia predominates and may result in structural remodeling and fibrosis [82–85] (Fig. 7).

**Fig. 8:**
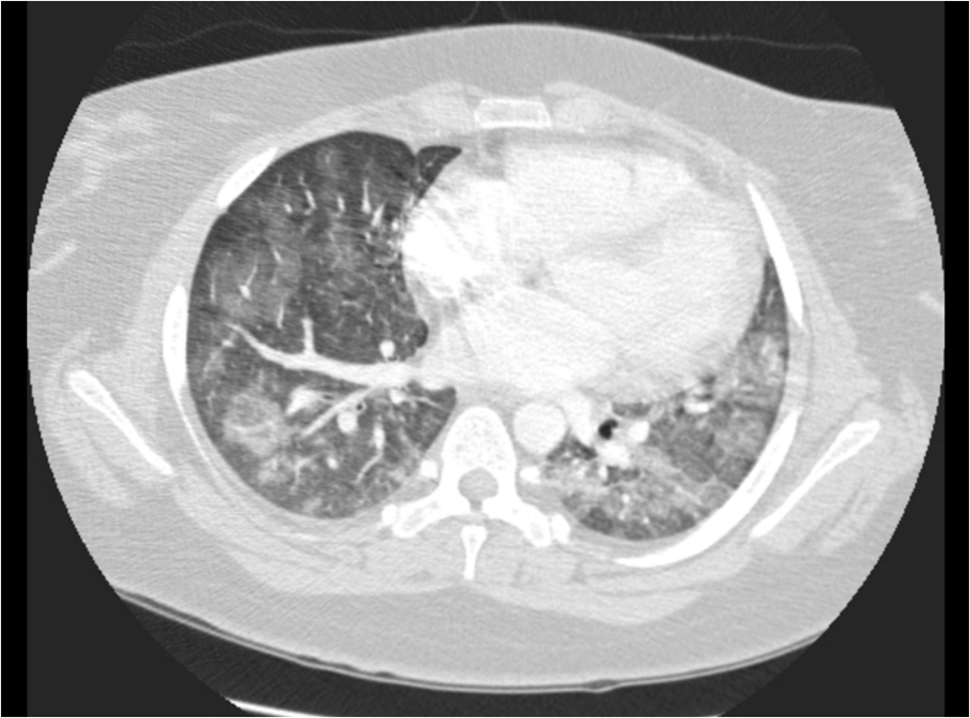
18-year-old female diagnosed with systemic lupus erythematosus when she was 16 years old presenting acutely. Axial CT image with intravenous contrast showing extensive bilateral ground-glass opacity and consolidation suggesting acute lupus pneumonitis

The major CT finding in the acute DAD phase is patchy, but typically extensive, geographic ground-glass opacity, often accompanied by interlobular septal thickening and areas of consolidation. These CT findings correspond to the airspace filling by cellular debris and exudative fluid as well as interstitial edema seen in the histopathology [74, 86] (Fig. 8). Dependent atelectasis is also commonly seen in the acute DAD stage [87]. In the organizing DAD phase, more widespread lung involvement will often be seen, with a greater degree of dependent-predominant consolidation and volume loss as well as traction bronchiectasis/bronchiolectasis [74, 88, 89]. Although the imaging features may ultimately resolve over time, fibrosis frequently remains that appears on CT as reticulation, parenchymal bands, architectural distortion, and traction bronchiectasis/bronchiolectasis [86, 90–92].

## Juvenile idiopathic arthritis

Juvenile idiopathic arthritis (JIA) is not a single entity, but a term encompassing all forms of arthritis of unknown origin that begin before a patient is 16 years of age and persist for more than 6 weeks [93, 94]. Systemic JIA is a category of JIA that tends to occur in young children and is characterized by arthritis and prominent systemic/extraarticular features including daily spiking fever, evanescent rash, generalized lymph node enlargement, hepatomegaly, splenomegaly, and/or serositis [94–97]. Although pleuritis/pleural effusion and pericarditis/pericardial effusion are the most common thoracic manifestations of systemic JIA, in the past decade there has been increasing recognition of a lung disease with high mortality in systemic JIA patients. Patients with systemic JIA that develop the lung disease tend to be young at systemic JIA onset (i.e., less than 3–5 years of age), and have a history of macrophage activation syndrome, reaction to cytokine-targeted biologics (particularly tocilizumab), and digital clubbing [8, 98].

### Pulmonary alveolar proteinosis and endogenous lipoid pneumonia

**Fig. 9:**
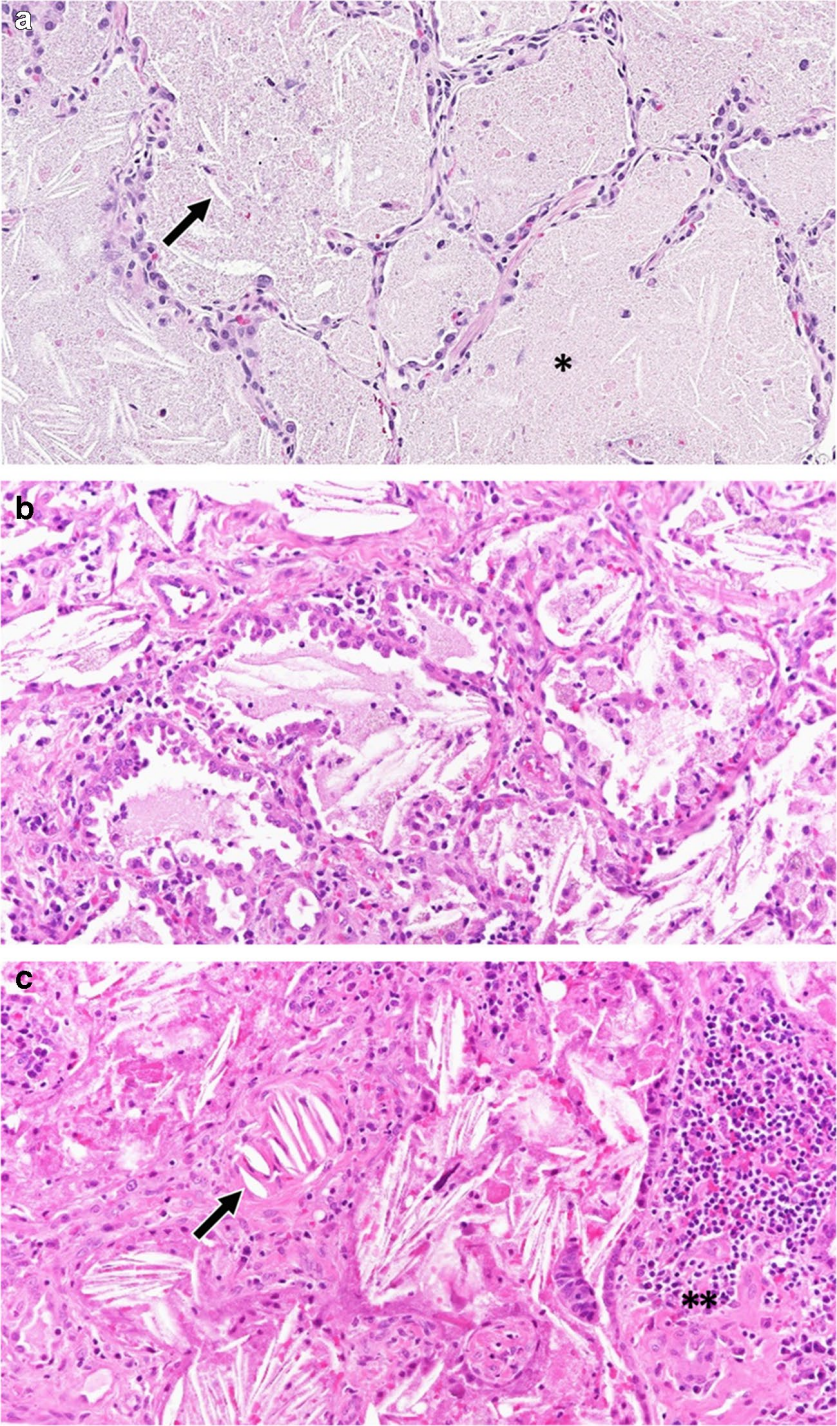
Histologic spectrum of systemic juvenile idiopathic arthritis-associated pulmonary alveolar proteinosis and endogenous lipoid pneumonia. **a** Pulmonary alveolar proteinosis with predominant proteinaceous material (*) with few cholesterol clefts (arrow) filling alveolar spaces (H&E, 200×). **b** Mixed features of pulmonary alveolar proteinosis and endogenous lipoid pneumonia (H&E, 200×). **c** Endogenous lipoid pneumonia with more abundant macrophages and cholesterol clefts than typically seen in pulmonary alveolar proteinosis along with an associated fibroinflammatory response (**) (H&E, 200×)

The histopathology of systemic JIA-associated lung disease lies on a spectrum of combined features of pulmonary alveolar proteinosis and endogenous lipoid pneumonia, a distinct pattern in rheumatic disease [8, 98]. The pulmonary alveolar proteinosis end of the spectrum is characterized by predominant distal airway and alveolar space filling with granular proteinaceous material with variable numbers of alveolar macrophages with vacuolated foamy cytoplasm and cholesterol clefts. The endogenous lipoid pneumonia end of the spectrum is typified by more abundant macrophages and multinucleated giant cells containing vacuoles both within airspaces and the interstitium. Cholesterol clefts are often more prominent and associated with a fibroinflammatory response (Fig. 9). Vasculopathy can also be seen in systemic JIA-associated lung disease including arterial hypertensive changes with arterial wall thickening.

**Fig. 10:**
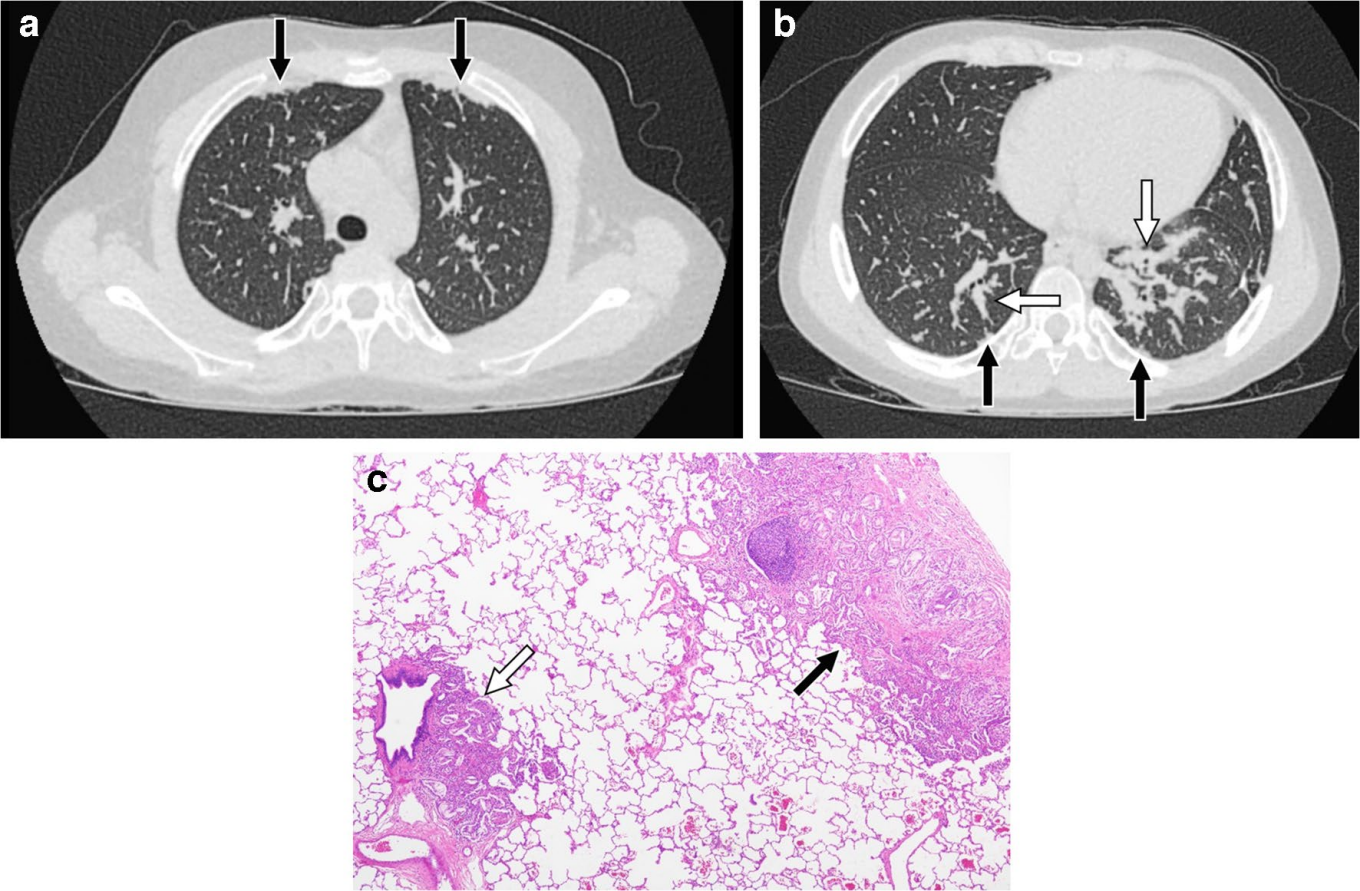
11-year-old male with systemic juvenile idiopathic arthritis. **a**, **b** Axial CT images without intravenous contrast showing mild subpleural consolidation in the anterior upper lobes and peripheral lower lobes (black arrows) as well as mild peribronchovascular consolidation in the lower lobes (white arrows). **c** Histologic image showing subpleural (black arrow) and peribronchiolar (white arrow) lung involvement (H&E, 40×)

**Fig. 11:**
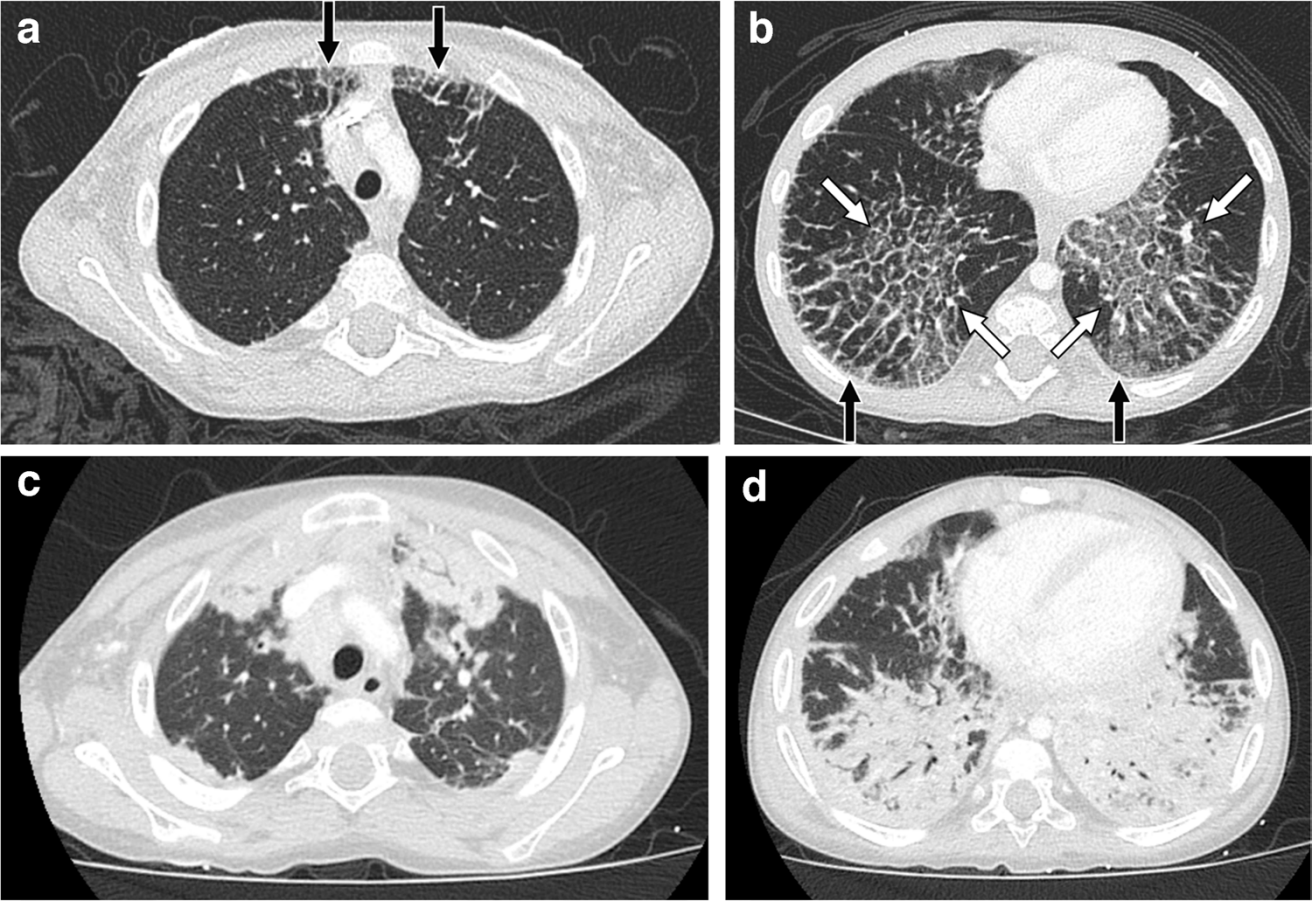
2-year-old male with systemic juvenile idiopathic arthritis. **a**, **b** Axial CT images with intravenous contrast showing ground-glass opacity, interlobular septal thickening, and intralobular lines in a crazy paving pattern in a subpleural distribution in the anterior upper lobes and peripheral lower lobes (black arrows), and in a peribronchovascular distribution in the lower lobes (white arrows). **c**, **d** Follow-up axial CT images with intravenous contrast at 5 years of age showing extensive consolidation at sites of prior ground-glass opacity and interlobular septal thickening

**Fig. 12:**
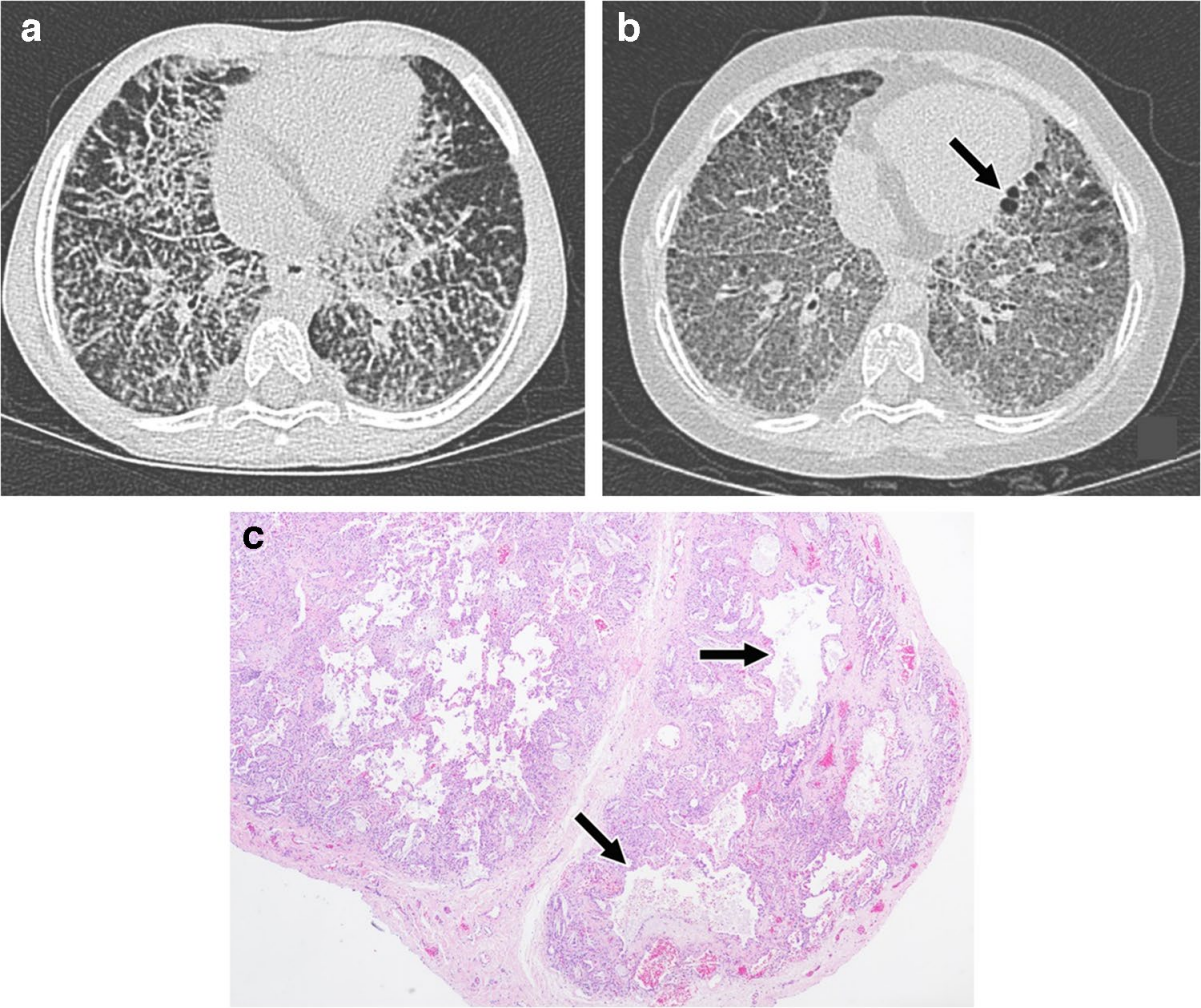
4-year-old female with systemic juvenile idiopathic arthritis. **a** Axial CT image without intravenous contrast showing widespread interlobular septal thickening. **b** Follow-up axial CT image without intravenous contrast at 5 years of age showing diffuse ground-glass opacity, new subpleural cysts (arrow), and less pronounced septal thickening. **c** Histologic image showing subpleural cystic spaces (black arrows) containing proteinaceous material (H&E, 40×)

On CT, systemic JIA-associated lung disease typically manifests as ground-glass opacity when at least partial aeration of airspaces remains in areas of lung involvement, consolidation when there is no remaining air in the airspaces, and/or interlobular septal thickening. Sometimes areas of ground-glass opacity have superimposed interlobular septal thickening and intralobular lines in a crazy paving pattern. Lung findings are typically seen in a subpleural and/or peribronchovascular distribution, although they can be diffuse. When subpleural involvement is seen it tends to involve the anterior upper lobes and peripheral lower lobes (Figs. 10 and 11). Subpleural predominant cysts can also be seen in patients with advanced disease [8, 99] (Fig. 12).

## Mixed connective tissue disease

Mixed connective tissue disease (MCTD) is characterized by a combination of features of systemic sclerosis, systemic lupus erythematosus, and dermatomyositis associated with a high titer of anti-U1 ribonucleoprotein antibodies [100, 101]. Among all MCTD cases, 23% begin in childhood [102]. MCTD is one of the least frequent rheumatologic diseases seen in the pediatric population, with a median age of onset of 11 years and a three times increased incidence in girls as compared to boys [103]. Typical clinical manifestations include arthritis, Raynaud phenomenon, swollen fingers and hands, myositis, esophageal dysmotility, fever, and skin changes that are seen with systemic sclerosis, SLE, and/or juvenile dermatomyositis [23, 101, 104, 105].

**Fig. 13:**
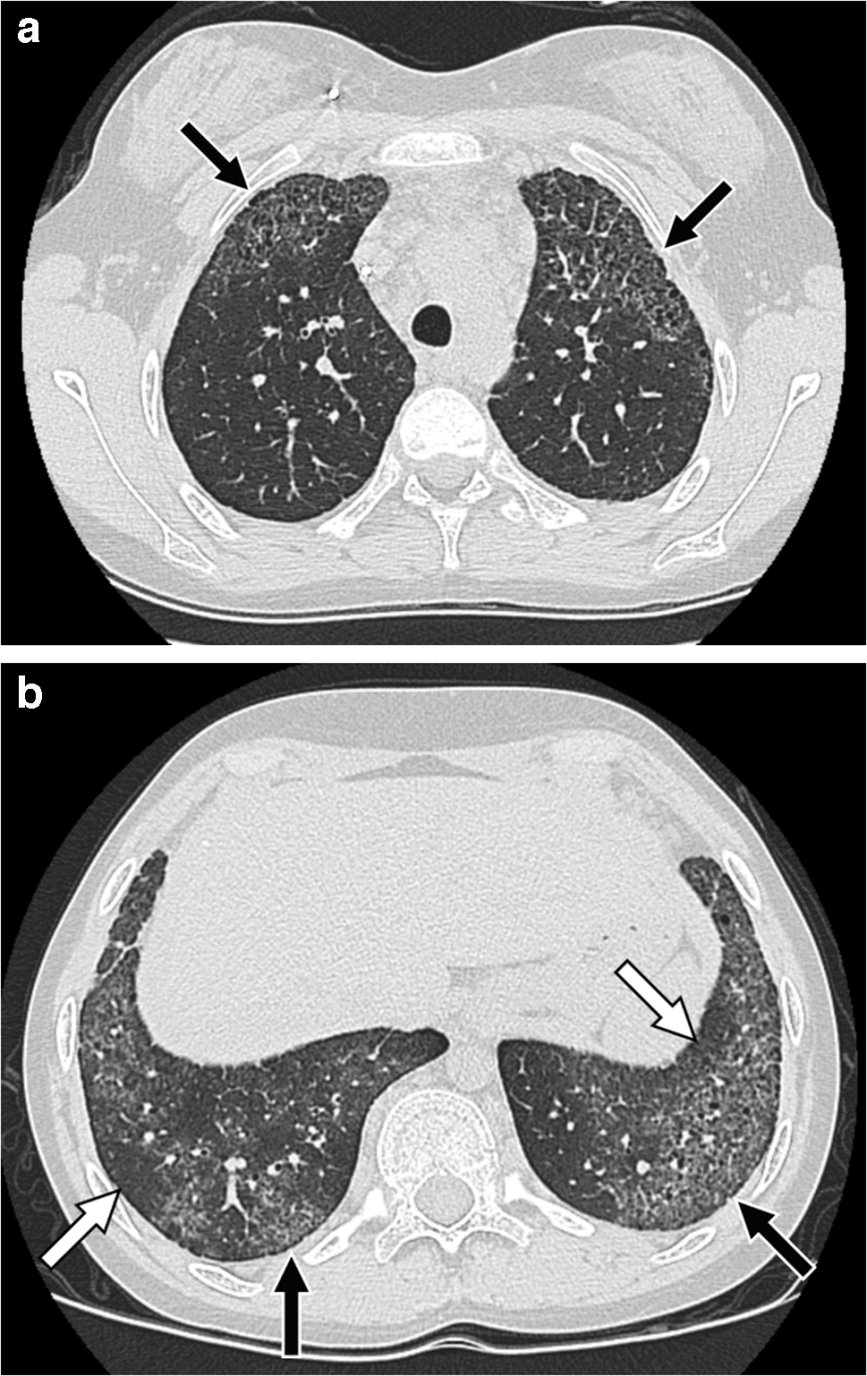
13-year-old female with mixed connective tissue disease. **a**, **b** Axial CT images without intravenous contrast showing ground-glass opacity, reticulation, and bronchiolectasis in the lower lobes and anterior upper lobes with areas of subpleural sparing in the lower lobes (black arrows) suggesting nonspecific interstitial pneumonia

Thoracic manifestations include a combination of features seen with systemic sclerosis, SLE, and juvenile dermatomyositis. Lung disease is common and typically manifests as NSIP, OP, or overlapping NSIP/OP patterns of disease [1, 10, 104, 106–111] (Fig. 13). Pleuritis and/or pericarditis in the presence or absence of effusions can also be seen [104, 112, 113]. Accompanying pulmonary hypertension is rare in children, but is a serious complication associated with increased mortality [101, 102, 104, 114]. Pulmonary arterial hypertension in the setting of MCTD pathologically manifests as pulmonary arterial intimal proliferation and medial hyperplasia that is accompanied by little or no interstitial fibrosis in the surrounding lung parenchyma [1, 102].

## Conclusion

Connective tissue disease-associated lung disease typically manifests as one of several patterns of lung disease, with certain lung disease patterns tending to occur in association with certain connective tissue diseases (Table 3). Knowledge of these lung disease patterns and the connective tissue diseases they are associated with can help the radiologist identify and better characterize connective tissue disease-associated lung disease. We conclude this article with a few key take-home points regarding connective tissue disease-associated lung disease in children:

**Table 3:** Key features of connective tissue disease-associated lung disease patterns

	Overview	Histopathology	CT features
Nonspecific interstitial pneumonia	• Highly associated with connective tissue disease • Can be seen with all connective tissue diseases • Most commonly seen with systemic sclerosis	• Spatially and temporally uniform alveolar septal chronic interstitial inflammation and/or fibrosis	• Lower lobe predominant ground-glass opacity • Reticulation • Traction bronchiectasis/bronchiolectasis • Subpleural sparing (not always seen, but suggestive of NSIP)
Lymphoid interstitial pneumonia	• Most commonly seen with Sjogren syndrome	• Densely cellular alveolar septal infiltrate composed of polyclonal lymphocytes and plasma cells	• Ground-glass opacity • Ill-defined centrilobular nodules • Interlobular septal and peribronchovascular thickening • Cysts
Organizing pneumonia	• Can be seen with all connective tissue diseases • Most commonly seen with juvenile dermatomyositis	• Fibroblastic tissue proliferations filling small airways and peribronchiolar alveolar spaces	• Peribronchovascular and peripheral consolidation • Perilobular opacities • Reversed halo/atoll sign
Diffuse alveolar damage	• Can be seen with juvenile dermatomyositis and SLE • High mortality	• Acute phase: hyaline membranes, edema, small arterial thrombi • Organizing phase: alveolar epithelial hyperplasia and metaplasia, fibroblast proliferation, structural remodeling, fibrosis	• Extensive ground-glass opacity • Dependent consolidation • Volume loss • Reticulation and traction bronchiectasis/bronchiolectasis with evolution
Pulmonary alveolar proteinosis/endogenous lipoid pneumonia	• Unique to systemic JIA among the connective tissue diseases	• Airspace filling by granular proteinaceous material, vacuolated macrophages and cholesterol clefts • Multinucleated giant cells with vacuoles and variable fibroinflammatory response	• Ground-glass opacity • Consolidation • Interlobular septal thickening • Subpleural and/or peribronchovascular distribution

*SLE* systemic lupus erythematosus, *JIA* juvenile idiopathic arthritis, *NSIP* nonspecific interstitial pneumonia

- Connective tissue disease-associated lung disease should be considered if any of the described CT patterns of lung disease are observed, even in patients without known connective tissue disease, with suspicion heightened if bilateral subpleural anterior upper lobe involvement is present or there are other thoracic manifestations associated with connective tissue disease.
- NSIP and organizing pneumonia can be seen in association with any of the connective tissue diseases.
- LIP is most commonly associated with Sjogren syndrome.
- A spectrum of pulmonary alveolar proteinosis and endogenous lipoid pneumonia is unique to systemic JIA among the connective tissue diseases.
- Diffuse alveolar damage can be seen in juvenile dermatomyositis and SLE and is associated with a high mortality rate.
- Alternative or superimposed processes such as infection, edema, medication-induced pulmonary injury, aspiration, and lymphoproliferative disease should be considered in addition to connective tissue disease-associated lung disease in patients with connective tissue disease.

## Data Availability

Not applicable.
